# Opportunistic QCT Bone Mineral Density Measurements Predicting Osteoporotic Fractures: A Use Case in a Prospective Clinical Cohort

**DOI:** 10.3389/fendo.2020.586352

**Published:** 2020-11-09

**Authors:** Yannik Leonhardt, Pauline May, Olga Gordijenko, Veronika A. Koeppen-Ursic, Henrike Brandhorst, Claus Zimmer, Marcus R. Makowski, Thomas Baum, Jan S. Kirschke, Alexandra S. Gersing, Vanadin Seifert-Klauss, Benedikt J. Schwaiger

**Affiliations:** ^1^ Department of Radiology, School of Medicine, Technical University of Munich, Munich, Germany; ^2^ Interdisciplinary Osteoporosis Center, Department of Gynaecology, School of Medicine, Technical University of Munich, Munich, Germany; ^3^ Department of Trauma Surgery, School of Medicine, Technical University of Munich, Munich, Germany; ^4^ Department of Orthopedics and Trauma Surgery, Klinikum Freising, Technical University of Munich, Freising, Germany; ^5^ Department of Neuroradiology, School of Medicine, Technical University of Munich, Munich, Germany

**Keywords:** osteoporosis, bone mineral density, QCT, DXA, fracture prediction, osteoporotic fracture, fracture liaison service

## Abstract

**Purpose:**

To assess whether volumetric vertebral bone mineral density (BMD) measured with opportunistic quantitative computed tomography (QCT) (i.e., CT acquired for other reasons) can predict osteoporotic fracture occurrence in a prospective clinical cohort and how this performs in comparison to dual-energy X-ray absorptiometry (DXA) measurements.

**Methods:**

In the database of our fracture liaison service, 58 patients (73 ± 11 years, 72% women) were identified that had at least one prevalent low-energy fracture and had undergone CT of the spine. BMD was determined by converting HU using scanner-specific conversion equations. Baseline DXA was available for 31 patients. During a 3-year follow-up, new fractures were diagnosed either by (i) recent in-house imaging or (ii) clinical follow-up with validated external reports. Associations were assessed using logistic regression models, and cut-off values were determined with ROC/Youden analyses.

**Results:**

Within 3 years, 20 of 58 patients presented new low-energy fractures (34%). Mean QCT BMD of patients with fractures was significantly lower (56 ± 20 vs. 91 ± 38 mg/cm^3^; p = 0.003) and age was higher (77 ± 10 vs. 71 ± 11 years; p = 0.037). QCT BMD was significantly associated with the occurrence of new fractures, and the OR for developing a new fracture during follow-up was 1.034 (95% CI, 1.010–1.058, p = 0.005), suggesting 3% higher odds for every unit of BMD decrease (1 mg/cm^3^). Age and sex showed no association. For the differentiation between patients with and without new fractures, ROC showed an AUC of 0.76 and a Youden’s Index of J = 0.48, suggesting an optimal cut-off value of 82 mg/cm^3^. DXA T-scores showed no significant association with fracture occurrence in analogous regression models.

**Conclusion:**

In this use case, opportunistic BMD measurements attained through QCT predicted fractures during a 3-year follow-up. This suggests that opportunistic measurements are useful to reduce the diagnostic gap and evaluate the fracture risk in osteoporotic patients.

## Introduction

Osteoporosis is a metabolic bone disease characterized by an overall reduced bone mass and microarchitectural deterioration of the bone tissue leading to reduced bone strength and low-energy fractures (1, 2). Complications such as pain and immobilization are not only a burden for the patient but also for health care and social security systems due to the ensuing financial costs (3). However, osteoporosis is a condition that can be treated (4). For this, the detection and assessment of osteoporosis at an early stage and evaluation of the risk for osteoporotic fractures is of high importance.

To optimize diagnosis and treatment of osteoporosis and to improve secondary prevention of fractures, fracture liaison services (FLS) are implemented in many hospitals; through a standardized intervention program, the FLS identifies patients with a high likelihood of osteoporosis and routes them to further diagnostics and adequate treatment (5–7). A pillar in this process is accurate imaging of osteoporosis and osteoporotic fractures.

Dual-energy X-ray absorptiometry (DXA) and dedicated quantitative CT (QCT) are currently the clinical standards for assessing osteoporosis by determining the bone mineral density (BMD) (3). However, those methods require specific examinations that are associated with additional radiation exposure and costs. Lack of access to those diagnostic tools and underutilization (e.g., due to patient noncompliance) create a diagnostic gap, where patients with prevalent osteoporotic fractures are not diagnosed with osteoporosis, which subsequently leads to a delay of adequate care. It has been reported that in patients who sustain a fragility fracture, fewer than 20% of individuals receive therapies to reduce the risk of future fractures within the year following the fracture (8).

In many cases, standard CT scans are already performed for fracture detection and characterization. Osteodensitometry, not only in such CT scans but also in routine CT scans that have been acquired for other purposes, can distinguish osteoporotic from healthy bone (9). Densitometry by opportunistic QCT, i.e., based on CT examinations that were acquired for other purposes such as morphological fracture assessment has a huge potential for screening patients at risk for osteoporosis (10, 11). The CT scans are either asynchronously calibrated (separate scans of calibration phantoms and patients) or tissue-calibrated (using tissue within the scan such as muscle and fat tissue that are known for little variance regarding density). Recently, osteoporotic trabecular BMD of lumbar vertebrae was assessed by opportunistic QCT and allowed for better risk assessment of imminent vertebral fractures than DXA in neurosurgical and oncologic patients (12).

In this study, we investigated the use of BMD measurement by above-mentioned asynchronously calibrated opportunistic QCT with regard to risk assessment and predictive value for osteoporotic fractures.

## Methods

### Study Population and Follow-Up

From 2015 to 2018 a total of 241 patients who had been hospitalized due to low-energy fractures of the spine, the proximal femur, the proximal humerus, or the distal radius were approached by the “Fracture Liaison Service” (FLS) in our hospital and provided informed consent. As an inclusion criterion for this study, patients had to undergo at least one CT including the lumbar spine either indicated for a suspected acute vertebral fracture or for other reasons during a visit at our hospital shortly before the baseline FLS visit, which applied to 79 patients. The initial indication for the majority of the CT imaging was fracture detection and characterization (41 out of the 58 ultimately included patients, 71%), while the rest was performed for other acute diagnostic purposes (e.g., suspected kidney stones or suspected small bowel obstruction; 8/58 patients, 14%) and routine examinations such as follow-up of abdominal aortic aneurysms (9/58 patients, 15%).

In a follow-up period of at least 3 years, we obtained information on the occurrence of new low-energy fractures in the above-named anatomical regions either by (i) new findings in imaging performed in our institute or by (ii) clinical follow-up in the osteoporosis center of our hospital with patient reporting and/or validated external reports of new vertebral fractures.

Of the 79 eligible patients, 21 had to be excluded for the following reasons: 14 had no sufficient follow-up (neither CT at our institution nor validated external reports), and in 7 patients BMD measurements in at least 3 thoracolumbar vertebrae in the baseline CT were impossible due to heavy impairment of the image quality (e.g., metal artifacts) or alterations in too many vertebrae (e.g., too many fractured vertebrae or vertebrae after kyphoplasty). Ultimately, 58 patients (73 ± 11 years, 72% women) were included in our study.

The study was approved by the local institutional review board (Ethics Commission of the Medical Faculty, Technical University of Munich, Germany; ethics approval number 27/19S).

### Computed Tomography

Five different multidetector computed tomography (MDCT) scanners in our hospital were used for the scans of the lumbar spine (IQon, Brilliance 64 and iCT 256 by Philips Medical Care; Somatom Definition AS+ and Definition AS by Siemens Healthineers), partly with administration intravenous contrast medium (Imeron 400, Bracco). The data was acquired in helical mode with a peak tube voltage of 120 kVp, a slice thickness of 0.9 to 1 mm, and adaptive tube load.

### Dual Energy X-ray Absorptiometry

For a subgroup analysis, we identified 31 patients of whom results from a baseline DXA analysis were additionally available. For all DXA measurements, one densitometer (GE Lunar Prodigy, GE Healthcare) was used. The measurements were performed by trained technologists and evaluated by experienced physicians, under the supervision of a certified densitometrist. Since this study focused on vertebral BMD, only measurements in lumbar vertebrae were considered to ensure comparability to QCT BMD measurements (see below). Fractured or otherwise altered vertebrae (e.g., vertebrae with severe degenerative changes, vertebrae after vertebro-/kyphoplasty) were excluded. Osteoporosis was defined as T ≤ − 2.5 standard deviations (SD), osteopenia as − 2.5 < T ≤ − 1 SD (13).

### Opportunistic QCT and BMD Measurement

**Figure 1:**
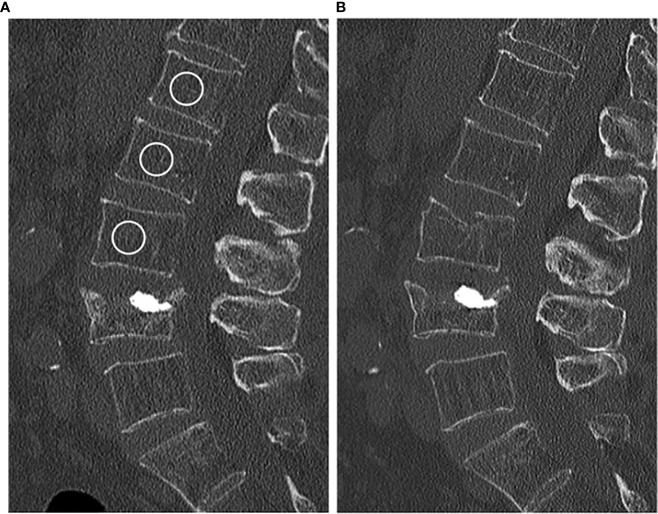
**(A)** Baseline CT scan of a 84-year-old female. The circles in vertebrae L1–3 represent the ROIs used for calculating the BMD (in this case 75.1 mg/cm^3^). **(B)** New vertebral compression fracture of L3 in the same patient 11 months later.

For Hounsfield unit (HU) measurements, a mid-line 15 mm MPR section in sagittal reformations was created with a PACS tool (IDS7, Sectra). Then, cylindrical volumes of interest were manually positioned in three non-fractured thoracolumbar vertebrae (ideally, L1–3) by one radiologist (YL), and mean HU was noted (**Figure 1**) (14). Fractured or otherwise altered vertebrae (e.g., vertebrae with severe degenerative changes, vertebrae after vertebro-/kyphoplasty) were not used for HU measurements. The HU values were then converted into BMD using HU-to-BMD conversion as previously described: (i) 0.928 × HU + 4.5 mg/cm^3^ for the IQon Spectral CT, (ii) 0.855 × HU + 1.172 mg/cm^3^ for the Philips iCT 256, (iii) 0.778 × HU − 4.693 mg/cm^3^ for the Philips Brilliance 64, (iv) 1.011 × HU − 3.385 mg/cm^3^ for the Siemens Somatom Definition AS+, and (v) 0.985 × HU + 15.516 mg/cm^3^ for the Siemens Somatom Definition (12, 15). A BMD correction offset for contrast-enhanced CT scans with arterial (-8.6 mg/cm^3^) and portal venous contrast phase (-15.8 mg/cm^3^) was added (16). Osteoporosis was defined as BMD < 80 mg/cm^3^ and osteopenia as 80 ≤ BMD ≤ 120 mg/cm^3^ (17).

### Statistical Analysis

Statistical analysis was performed with SPSS version 25 software (IBM, New York, USA). Differences in baseline characteristics of patients with and without fractures during follow-up were assessed by *t-*tests for continuous variables and chi-square test for categorical variables. Associations of patient characteristics (age and sex) and BMD with the occurrence of follow-up fractures were analyzed by logistic regression analysis, separately for QCT BMD and DXA T-scores; the *goodness of fit* for the regression model was evaluated by Nagelkerke R^2^ and the effect size according to Cohen (18). To assess optimal cut-off values for QCT BMD, receiver operating characteristic (ROC) with Youdens J statistic was performed. Significance was assumed if α-level was p < 0.05.

## Results

**Figure 2:**
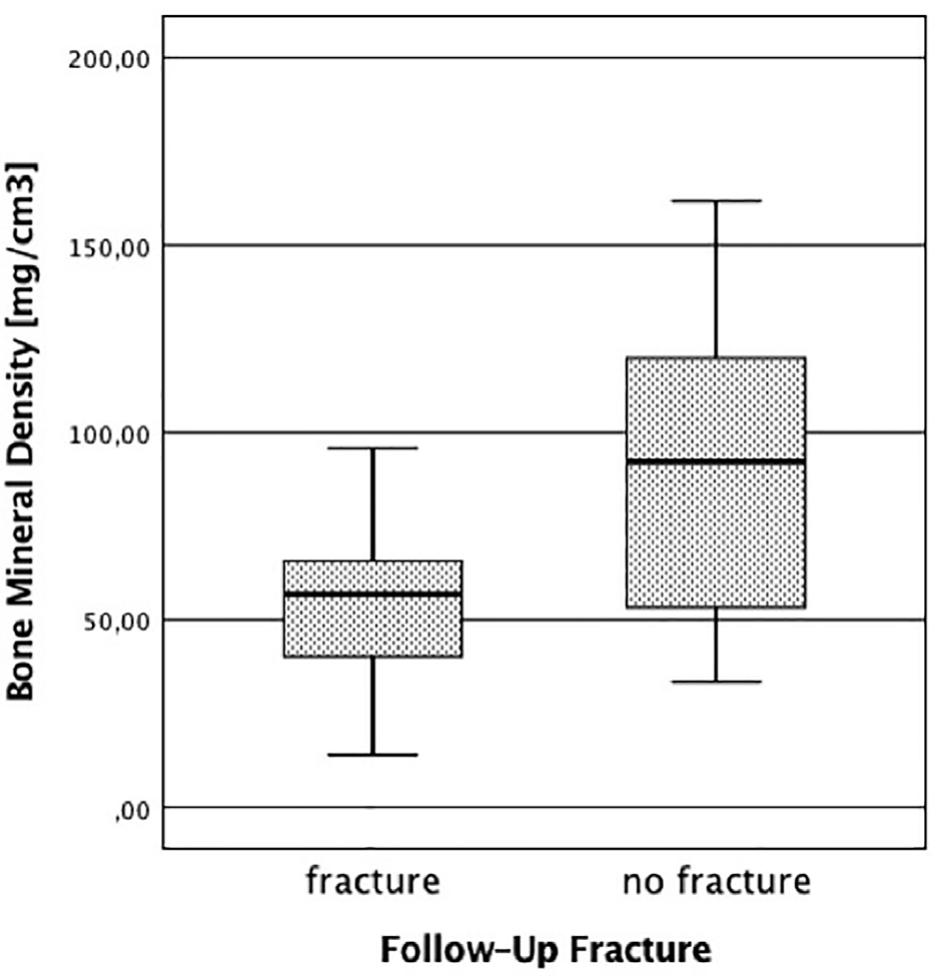
Bone mineral density in patients with and without follow-up fracture. The BMD was calculated using the initial baseline CT scans.

We identified 58 patients in our FLS database that met the criteria specified above for study participation (i.e., sufficient baseline image quality and complete follow-up information). Of the 58 patients, 20 reported at least one new low-energy fracture at any site up to the 3-year-follow-up. As shown in **Table 1**, patients with new low-energy fractures during follow-up were significantly older with a mean age of 76.71 ± 9.61 years and had a significantly lower mean QCT BMD with 56.19 ± 19.96 mg/cm^3^ compared to patients without fracture with a mean age of 70.63 ± 10.64 years and a mean QCT BMD of 90.71 ± 37.82 mg/cm^3^ (p < 0.05; **Figure 2**).

**Table 1:** Characteristics of patients with and without new low-energy fractures during the 3-year follow up.

	total (n = 58)	no fracture (n = 38)	fracture (n = 20)	p-value
Age (years)	72.80 ± 10.69	70.63 ± 10.64	76.71 ± 9.61	0.037*
Sex (*n)*				0.300
Male	16 (28%)	12 (32%)	4 (20%)	
Female	42 (72%)	26 (68%)	16 (80%)	
BMD by QCT (mg/cm^3^)	78.42 ± 36.55	90.71 ± 37.82	56.19 ± 19.96	0.003*
Densitometry by DXA (*n*)	31	22	9	
DXA T-score	-2.00 ± 1.65	-1.66 ± 1.75	-2.83 ± 0.98	0.078
Bone density by CT (*n)*				
Normal	9 (15%)	9 (24%)	0 (0%)	
Osteopenia	16 (28%)	13 (34%)	3 (15%)	
Osteoporosis	33 (57%)	16 (42%)	17 (85%)	
Bone density by DXA (*n*)				
Normal	7 (23%)	7 (32%)	0 (0%)	
Osteopenia	13 (42%)	10 (45%)	3 (33%)	
Osteoporosis	11 (35%)	5 (23%)	6 (67%)	

* significant at an α-level of 0.05.

Comparing the BMD calculated by CT, no patient in the fracture-group (n = 20) had QCT BMD values over 120 mg/cm^3^ and almost all patients (17 out of 20) had QCT BMD values indicating osteoporosis (<80 mg/cm^3^); in the no fracture-group (n = 38), 9 patients had QCT BMD values over 120 mg/cm^3^, 13 patients were osteopenic, i.e., between 80 and 120 mg/cm^3^, and 16 patients were osteoporotic below 80 mg/cm^3^.

Logistic regression analysis was performed to analyze associations of QCT BMD, age, and sex with the occurrence on new fractures in our patient population (**Table 2**). QCT BMD was significantly associated with the occurrence of new fractures, and the OR for developing a new fracture during follow-up was 1.034 (95% CI, 1.010–1.058, p = 0.005), suggesting 3% higher odds for every unit of BMD decrease (1mg/cm^3^); the overall regression model was significant (p = 0.002) and had a strong effect size (f = 0.66). Age and sex showed no association.

**Table 2:** Associations of age, sex, and BMD with the occurrence of follow-up fractures analyzed by logistic regression analysis.

	β	p-value	Odds ratio [Exp(β)]	95% CI
BMD by CT	0.33	0.005*	1.034	[1.010; 1.058]
Age	-0.28	0.443	0.973	[0.906; 1.044]
Sex	0.487	0.580	1.627	[0.290; 9.143]
Constant	-0.117	0.968	0.890	

Significant regression model [χ^2^(3) = 14.816, p = 0.002], Nagelkerke R^2^ = 0.305, strong effect size f = 0.66.

* significant at an α-level of 0.05.

**Figure 3:**
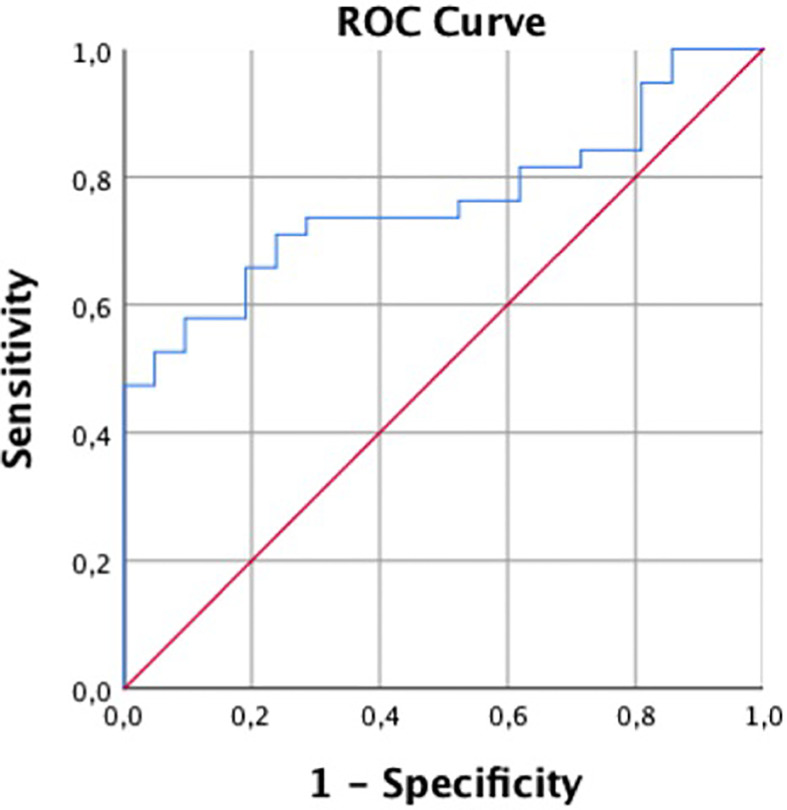
Receiver operating characteristic curve for predicting follow-up fractures by QCT.

For the differentiation of patients with and without incidental fractures, ROC showed an AUC of 0.76 and a Youden’s Index of J = 0.48, suggesting an optimal cut-off value of 82 mg/cm^3^, which almost equals the established cut-off for osteoporosis in QCT (**Figure 3**).

Of the patients, 31 had DXA (mean 118.68 ± 117.06 days after baseline CT); the mean T-scores of patients with follow-up fracture (n = 9, T-score = -2.83 ± 0.98) and without fracture (n = 22, T-score = -1.66 ± 1.75) did not differ significantly (p = 0.078). Pearson’s *r* showed a moderate correlation between QCT BMD and T-score (r=0.49). Comparing the DXA T-score, no patient in the fracture-group (n = 9) had a T-score above -1.0, 3 patients had osteopenic T-scores between -1.0 and -2.5, and six patients were classified as osteoporotic with T-scores below -2.5. In the group without fractures (n = 22), 7 patients had T-scores over -1.0, 10 patients between -1.0 and -2.5, and five patients were below -2.5 (**Table 1**).

## Discussion

In our prospective cohort, opportunistic BMD measurements, i.e., using CT examinations acquired for other purposes, were able to predict new low-energy fractures during a 3-year follow-up period. In contrast, no significant associations between DXA T-scores and the occurrence of new fractures were found.

Assessing osteoporosis and predicting osteoporotic fractures has been explored in previous studies analyzing and comparing different methods like DXA, dedicated QCT, standard CT, CT-based finite element analysis or, more recently, dual-layer spectral CT (15, 19–21). Previous studies have shown that simple lumbar vertebral trabecular attenuation measurements in CT examinations that were acquired for different purposes are a potentially valid method to predict future osteoporotic fractures (22). Löffler et al. for example showed in their retrospective study that osteoporotic trabecular BMD of lumbar vertebrae assessed by asynchronously calibrated opportunistic QCT was associated with an increased risk of incident vertebral fractures and allowed for better risk assessment of imminent vertebral fractures than dedicated DXA in mainly neurosurgical and oncologic patients (12).

The patients in our study were recruited through the “Fracture Liaison Service” (FLS) in our hospital, which was a clinical cooperation project evaluating low energy fractures, providing a diagnostic work-up for osteoporosis and initiating anti-osteoporotic treatment to prevent subsequent fractures (5). Therefore, our patient population represents a “real life” sample of patients with prevalent frailty fractures and at least clinically suspected osteoporosis at the time of inclusion. Interestingly, even in this high-risk cohort, there were many patients that never underwent a BMD measurement. This may be due to lack of access or availability of measurement slots or due to lack of patient compliance (intentional or due to other severe illness) and illustrates the “diagnostic gap” that is a substantial issue in the management of patients with osteoporosis.

It has been suggested that opportunistically using existing imaging data could reduce this diagnostic gap. As our data shows, (i) the majority of patients included in the study indeed did not have a dedicated BMD measurement and (ii) opportunistic measurements predicted new fractures during a 3-year follow-up. Interestingly, the odds for developing a fracture *increased* by 3% per one unit *decrease* of BMD by 1 mg/cm^3^.

The mean age was significantly higher in patients with a new fracture, which is in line with the literature (12). However, this effect was neutralized in logistic regression models, suggesting a collinearity between age and BMD.

31 patients also had osteodensitometry by DXA. While the absolute values of the T-scores were lower in patients with follow-up fractures, the difference was not statistically significant both in a *T* test in a logistic regression model.

Every patient that had T-scores indicating osteoporosis had QCT BMD with values below 80 mg/cm^3^ as well. On the other hand, eight patients showed osteoporotic QCT BMD values but had DXA T-scores above -2.5; out of those eight patients, two had follow-up fractures. This is in line with findings in other studies, where DXA struggled to correctly diagnose up to every second patient with manifest osteoporosis (23, 24). This discrepancy indicates that QCT BMD measurement might be a more sensitive diagnostic tool with value in addition to conventional methods like DXA.

This study has limitations. Since many patients included in the FLS database are treated near to their homes, not all underwent follow-up imaging at our institution. Therefore, in a subgroup of patients we had to rely on patient reporting and external reports diagnosing new fractures during follow-up. Therefore, e.g., subclinical vertebral fractures may have been missed, and the total number of fractures may have been underestimated in this study.

Furthermore, the subgroup of patients in which both opportunistic QCT and DXA measurements were available was small. Thereby the statistical power is weaker, which may be the reason why T-scores were not significantly associated with the occurrence of new fractures. Finally, the number of male patients included in this study, particularly in the fracture group, was relatively low, which warrants caution when applying the results to male patients. However, since this was a retrospective analysis, we were not able to adjust the sex ratio without excluding further patients, and moreover, this setting represents the clinical reality in which more female patients are seen with osteoporotic vertebral fractures.

In summary, opportunistic QCT BMD measurements were a valid tool to predict osteoporotic fractures during a three-year follow-up. CT is readily available for the imaging of suspected vertebral fractures in most hospitals, while in-house possibilities for bone mineral density measurements such as DXA and dedicated QCT may not be ubiquitous. Therefore, using this already existing imaging data for BMD measurements could be highly useful to minimize the diagnostic gap in patients with no access to bone density scanning despite prevalent fractures and high risk of further fractures and to support earlier therapeutic decisions.

## Data Availability Statement

The raw data supporting the conclusions of this article will be made available by the authors, without undue reservation.

## Ethics Statement

The studies involving human participants were reviewed and approved by Ethics Commission of the Medical Faculty, Technical University of Munich. The patients/participants provided their written informed consent to participate in this study.

## Author Contributions

YL: Methodology, formal analysis, investigation, data curation, writing (original draft), visualization, and project administration. PM: Methodology, formal analysis, investigation, data curation, visualization, project administration, writing (review and editing). OG, VK-U, and HB: Validation and writing (review and editing). CZ: Writing (review and editing), resources, and supervision. MM: Funding acquisition, writing (review and editing), resources, and supervision. TB: Conceptualization and writing (review and editing). JK: Conceptualization and writing (review and editing). VS-K: Methodology, writing (review and editing), project administration, and conceptualization. AG: Methodology, verification, and writing (review and editing). BS: Conceptualization, methodology, writing (review and editing), supervision, and project administration. All authors contributed to the article and approved the submitted version.

## Funding

JK has received research funding from the German Research Foundation (Deutsche Forschungsgemeinschaft, DFG; project 432290010) and the European Research Council (ERC) under the European Union’s Horizon 2020 research and innovation programme (637164 — iBack — ERC-2014-STG) as well as from the Nvidia Corporation. The funders were not involved in the study design, collection, analysis, interpretation of data, the writing of this article or the decision to submit it for publication.

## Conflict of Interest

The authors declare that the research was conducted in the absence of any commercial or financial relationships that could be construed as a potential conflict of interest.
